# CD36 Participates in PrP_106–126_-Induced Activation of Microglia

**DOI:** 10.1371/journal.pone.0030756

**Published:** 2012-01-26

**Authors:** Mohammed Kouadir, Lifeng Yang, Rongrong Tan, Fushan Shi, Yun Lu, Siming Zhang, Xiaomin Yin, Xiangmei Zhou, Deming Zhao

**Affiliations:** State Key Laboratories for Agrobiotechnology, National Animal Transmissible Spongiform Encephalopathy Laboratory, College of Veterinary Medicine, China Agricultural University, Beijing, China; Massachusetts General Hospital and Harvard Medical School, United States of America

## Abstract

Microglial activation is a characteristic feature of the pathogenesis of prion diseases. The molecular mechanisms that underlie prion-induced microglial activation are not very well understood. In the present study, we investigated the role of the class B scavenger receptor CD36 in microglial activation induced by neurotoxic prion protein (PrP) fragment 106–126 (PrP_106–126_). We first examined the time course of CD36 mRNA expression upon exposure to PrP_106–126_ in BV2 microglia. We then analyzed different parameters of microglial activation in PrP_106–126_-treated cells in the presence or not of anti-CD36 monoclonal antibody (mAb). The cells were first incubated for 1 h with CD36 monoclonal antibody to block the CD36 receptor, and were then treated with neurotoxic prion peptides PrP_106–126_. The results showed that PrP_106–126_ treatment led to a rapid yet transitory increase in the mRNA expression of *CD36*, upregulated mRNA and protein levels of proinflammatory cytokines (IL-1β, IL-6 and TNF-α), increased iNOS expression and nitric oxide (NO) production, stimulated the activation of NF-κB and caspase-1, and elevated Fyn activity. The blockade of CD36 had no effect on PrP_106–126_-stimulated NF-κB activation and TNF-α protein release, abrogated the PrP_106–126_-induced iNOS stimulation, downregulated IL-1β and IL-6 expression at both mRNA and protein levels as well as TNF-α mRNA expression, decreased NO production and Fyn phosphorylation, reduced caspase-1 cleavage induced by moderate PrP_106–126_ –treatment, but had no effect on caspase-1 activation after treatment with a high concentration of PrP_106–126_. Together, these results suggest that CD36 is involved in PrP_106–126_-induced microglial activation and that the participation of CD36 in the interaction between PrP_106–126_ and microglia may be mediated by Src tyrosine kinases. Our findings provide new insights into the mechanisms underlying the activation of microglia by neurotoxic prion peptides and open perspectives for new therapeutic strategies for prion diseases by modulation of CD36 signaling.

## Introduction

Prion diseases are a group of transmissible fatal neurodegenerative disorders of humans and animals, including bovine spongiform encephalopathy (BSE) in cattle, scrapie in sheep, and Creutzfeldt-Jakob disease (CJD) in humans. They are caused by the conversion of cellular prion protein (PrP^C^) into the pathological isoform (PrP^Sc^) through conformational changes [1], [2]. Neuropathological features of prion diseases include neuronal vacuolation, neuronal loss, astrogliosis and accumulation of activated microglial cells in affected brain areas [3].

Microglia, the resident macrophages of the central nervous system parenchyma, are exquisitely sensitive to pathological tissue alterations, altering their morphology and phenotype to adopt a so-called activated state and perform immunological functions in response to pathophysiological brain insults [4], [5]. A wealth of data now demonstrate that the microglia have very diverse effector functions, in line with macrophage populations in other organs [6]. Mounting evidences also indicate that microglial activation contributes to neuronal damage in several neurodegenerative diseases including Alzheimer's disease, prion diseases, Parkinson's disease, multiple sclerosis, and Huntington's disease [5], [7]. In prion diseases and other neurodegenerative disorders, microglia can become overactivated and release ROS, NO, and cytokines, which might cause vascular damage in addition to neurodegeneration [7], [8], [9], [10]. Pattern recognition receptors expressed on the microglial surface, including Toll-like receptors and scavenger receptors seem to associate physically to form a receptor complex, which is one of the primary, common pathways through which diverse toxin signals are transduced into ROS production in microglia [4], [11].

Scavenger receptor CD36 is a cell surface protein that is differentially regulated in microglia through development and in response to disease,and is known to be involved in microglia-mediated immune response in the central nervous system [12], [13]. The role of CD36 in the amyloid-beta (Aβ)-induced microglial activation in Alzheimer's disease has been extensively investigated [11], [14], but there has been no report so far of its role in prion diseases.

Neurotoxic prion protein (PrP) fragment 106–126 (PrP_106–126_) possesses similar physicochemical and pathological properties to PrP^Sc^, in that it forms amyloid fibrils with high β-sheet content, shows partial proteinase K resistance, and is neurotoxic in vitro [15]. Therefore, PrP_106–126_ is commonly used as a model for the investigation of PrP^Sc^ neurotoxicity.

In the present study, we investigated the role of the class B scavenger receptor CD36 in the activation of murine microglia induced by PrP_106–126_. The results of this study suggest that CD36 participates in PrP_106–126_-induced microglial activation by mediating iNOS, and pro-inflammatory cytokines up-regulation through the activation of Src tyrosine kinases.

## Materials and Methods

### Isolation and culture of microglia cells

Primary microglial cell cultures were obtained from neonatal Wistar rats 5–7 days old, according to the method described by Garcao et al. [9]. After sterilization with 75% ethanol for 3–5 min, the brain was dissected and the meninges were carefully removed under a stereomicroscope. Cerebral cortexes were collected and the tissue was dissociated by pipetting up and down for approximately 10 times, and then digested with trypsin (0.125%) for 15–20 min at 37°C, which was terminated with DMEM containing serum. The digested tissue was repeatedly pipetted to obtain the maximum number of single cells [16]. Then the cells were passed through a 200 µm mesh to obtain a single-cell suspension. After centrifugation at 100 g for 5 min and the cellular pellet was resuspended in DMEM containing 10% FCS, penicillin and streptomycin, and then plated in a 35 mm plastic tissue culture flask [17]. The mixed glia cells were cultured for about 6–7 days. The cells were suspended by agitation for 12 h on a rotary shaker (180 rpm) at 37°C and transferred to another flask. After incubation for 2 h at 37°C, microglia that selectively adhered to the plastic flask were washed with Ca2+/Mg2+-free PBS and grown in a serum-free medium before treatment with PrP_106–126_ (see below). The purity of the microglia cells was approximately 90% determined using anti-CD-11b antibodies.

BV2 cells, a murine microglia cell line, were obtained from Xiehe Medical University (Beijing, China).Cells were maintained in a 95% air, 5% CO _2_ atmosphere in DMEM-F12 supplemented with 10% fetal bovine serum (FBS; GIBCO), 100 µg/mL streptomycin, 100 U/mL penicillin (GIBCO), and 2 mM glutamine permilliliter.

#### Prion peptides

PrP peptides PrP_106–126_ and scrambled PrP_106–126_ (Scr-PrP) with sequence of KTNMKHMAGAAAAGAVVGGLG and AVGMHAGKGLANTAKAGAMVG were synthesized by Sangon Bio-Tech. The peptides were dissolved in Phosphate Buffer Solution (PBS) at a concentration of 2 mM and stored at −20°C as the stock solutions. The purity of prion peptides was >95% according to the synthesizer's data. The peptide solution (500 µM) in PBS (pH 7.4) was aged for 2–3 days at 37°C before use to increase fibrillogenicity of the peptide [16].

#### Reagents

The rabbit anti-mouse NF-κB p65, CD36, NOS-2 (i-NOS), caspase1, and p-Fyn antibodies were acquired from Beyotime Biotechnology (China), Gene Tex (Texas, USA), Hantian Biotechnology (China), Wuhan Boster Biotech (Wuhan, China), and Bioworld Technology (USA), respectively. Anti-mouse β-actin, and Max antibody were purchased from Beyotime Biotechnology (China). ELISA kits for mouse interleukins and Fast Protein Precipitation and Concentration KIT were purchased from Wuhan Boster Biotech (Wuhan, China). Reagents and apparatus used in immunoblotting assays were obtained from Bio-Rad (Hercules, CA). Alkaline phosphatase-linked anti-rabbit secondary antibody was from Beyotime Biotechnology (China).

#### Peptide treatment

Prion peptide 106–126 was aggregated for 72 h at 37°C in DMEM-F12 medium. Microglia were exposed or not to 1 µg/ml anti-CD36 antibody for 1 h and then treated with the aggregated peptide PrP_106–126_ in culture medium. Scr-PrP was used as a negative control. Three wells were used in each group of experimental conditions.

#### Extraction of nuclear and cytoplasmic protein and Western blotting

After cells treatment with 50 µM or 100 µM PrP_106–126_ for 12 or 24 h, the culture medium was discarded and cytoplasmic and nuclei proteins were extracted using Extract kit (Beyotime Biotechnology Inc., China). Equal amounts of protein were separated by SDS-PAGE on 8% gels, and the separated proteins were transferred onto a nitrocellulose membrane. Nonspecific binding sites were blocked by incubating the membrane with 5% fat-free dried milk in TBST (10 Mm TrisHC1, pH 7.5, 0.15 M NaCl, 0.05% Tween20) or bovine serum albumin (during the assessment of p-Fyn levels). Rabbit anti NF-κB p65 (1∶500), anti-iNOS (1∶200), anti-caspase1 (1∶100), or anti-p-Fyn (1∶500) antibodies were added and incubated for 1 h at 4°C. Membranes were washed with TBST and then incubated with the secondary antibody, an anti-rabbit horseradish peroxidase-conjugated goat antiserum (1∶5000). Bands of immuno-reactive protein were visualized, after membrane incubation with ECF reagent for 5 min, on a Versadoc image system. The blot was stripped and reprobed with antiactin antibody (for cytoplasmic extracts) or anti-Max (for nuclear extracts) to estimate the total amount of protein loaded in gel.

#### Cytokine Release Assessment

The levels of interleukin-1β (IL-1β), IL-6, TNF-α and i-NOS were determined in culture supernatants of BV2 microglia cells (0.2×10^6^ cells/cm2) treated for 12 h with the peptides(50 µM), anti-CD36 mAb (1 µg/ml), or combination of both of them by using enzyme-linked immunosorbent assay (ELISA) kits specific for mouse IL-1β, IL-6, TNF-α and i-NOS. The samples of culture supernatants, controls, and standards were first treated by Fast Protein Precipitation and Concentration KIT (Wuhan Boster Biotech, China) to increase protein concentration before ELISA analysis. Samples were then pipetted into microplates of these ELISA kits, according to the manufacturer's instructions (R&D Systems). The experiments were performed in duplicate, in four to six independent cell preparations. The absorbance was measured at 450 nm, with the correction wavelength at 540 nm, using microplate reader. The values were read off the standard curve and expressed as nanograms per liter (IL-1β, IL-6, TNF-α) or U/L(i-NOS).

#### Nitrite assay

Nitric oxide (NO) production was determined by the nitrite (NO2−) assay with the Griess reagent (0.1% N-(1-naphtyl)ethylenediamine dihydrochloride, 1% sulphanilamide and 2% phosphoric acid).The assay is based on the measurement of NO2−, a stable endproduct of the reaction between NO and molecular oxygen. Primary microglia were first pre-incubated or not with Anti-CD36mAb (1 µg/ml) or irrelevant rabbit IgG (Ab) and then treated for 12 hours with PBS, 50 µM PrP_106–126_ (PrP), or scrambled PrP106–126. Culture supernatants (50 µl) were mixed with an equal volume of Griess reagent for 15 min in the dark at room temperature, according to the method described by Huygen [18]. Absorbance was measured at 550 nm with a microplate reader. The nitrite concentration was determined from a sodium nitrite standard curve ranging from 0 to 100 µM. All experiments were performed in duplicate, in four to six independent cell preparations.

#### Real-time quantitative RT-PCR analysis of the expression of CD36 and proinflammatory cytokines

To determine the mRNA expression of *CD36* and proinflammatory cytokines, real-time PCR was carried out. Endogenous house-keeping gene β-actin was used as cDNA template control. PCR primers were used for amplification of genes cloned for real-time PCR (see Table 1). For construction of a standard curve, amplified fragments of target genes were cloned into pGEM-T-Vector by T-A clone technique, and five 10-fold serial dilutions of plasmid DNA ranging from 10^2^ to 10^6^ molecules were then prepared. Real-time quantitative PCR was performed using a DNA Engine Opticon™ 2 system (MJ Research, USA) and DyNAmo™ SYBR@ Green quantitative PCR kit (MJ Research, Waltham, MA, USA). The primers were used to amplify cDNA of the biological sample, negative controls and five plasmid DNA standards. PCR reaction system was carried out in a real-time PCR tube. The total volume was 20 µl, which includes 8.75 µl water, 0.3 µl of each primer (10 pmol), 10 µl Master Mix and 25 ng of reverse transcribed total RNA or plasmid DNA of the respective concentration. The PCR amplification was as follows: after denaturation at 94°C for 5 min, 36 PCR cycles were performed including 94°C for 30 s, 62°C for 20 s, 72°C for 20 s, and 1 s at 84°C appended for a single fluorescence measurement above melting temperature of possible primer-dimers. Finally a melting step was performed consisting of 10 s at 65°C and slow heating with a rate of 0.1°C per second up to 95°C with continuous fluorescence measurement. The mRNA levels of *CD36* and proinflammatory cytokines were calculated using an absolute standard curve method [19], [20]. The cDNA samples were amplified in parallel with plasmid standards in each run and their Ct values were plotted together with the standard curves, from which the normalized mRNA copy numbers were determined. All samples were analyzed in triplicate.

**Table 1:** Primers used for real-time PCR and cloning.

Genes	Primer sequences (5′-3′)	PCR product size	Annealing temperature	Genbank accession no.
*β-actin*	TGCTGTCCCTGTATGCCTCTG	223 bp	60°C	NM_007393
	TTGATGTACCGCACGATTTCC			
*CD36*	TCGGAACTGTGGGCTCATTG	294 bp	65°C	NM_001159555.1
	CCTCGGGGTCCTGAGTTATAT TTT C			
*IL-1β*	GCCTCAAAGGAAAGAATCTA	297 bp	47°C	NM_008361
	AAACAGTCCAGCCCATACTT			
*IL6*	TGCCTTCTTGGGACTGAT	384	55°C	NM_031168
	CTGGCTTTGTCTTTCTTGTT			
*TNF-α*	GGCGGTGCCTATGTCTCA	221 bp	55°C	NM_013693
	CACTTGGTGGTTTGCTACG			

#### Statistical analysis

Differences between groups were assessed by one-way ANOVA and post hoc Turkey's test using SPSS 13.0 software (USA). Data are expressed as means ± S.D. Differences with P<0.05 were considered statistically significant.

## Results

### 1- PrP_106–126_ induced an increase of *CD36* mRNA levels in BV2 microglia

**Figure 1:**
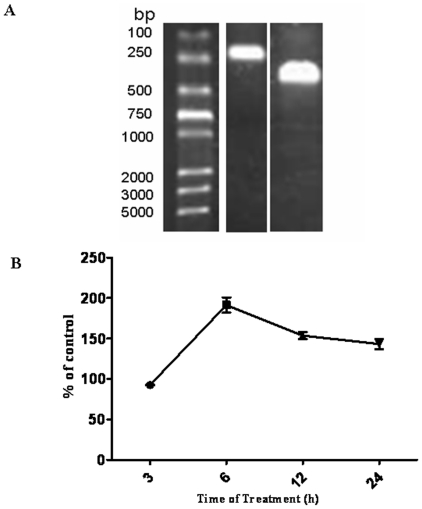
Quantitative RT-PCR analysis of PrP106–126 effect on mRNA expression of *CD36*. A. Electrophoresis of RT-PCR-amplified CD36 (294 bp) and β-actin (223 bp) on 8% agarose gel and stained with ethidium bromide. B. Time course of CD36 mRNA Expression upon exposure to PrP_106–126_ in BV2 microglia. Cells were treated for 3, 6, 12 h, or 24 hours with 100 µM PrP_106–126_. Total mRNA was isolated and reverse-transcribed. The mRNA level of CD36 were measured by quantitative RT-PCR. Expression of a receptor at each time point is expressed as a percentage of expression in control cells exposed to 100 µM scrambled PrP_106–126_ only for the same time. All data are mean ± s.d. of triplicate samples and are representative of an experimental n of 3 or 4. *, P<0.05.

BV2 cells were incubated with 100 µM of neurotoxic PrP_106–126_ or Scr-PrP for 3, 6, 12, and 24 h. RNA aliquots extracted from treated BV2 cells were reverse transcribed as described in Materials and Methods, and the PCR was used to amplify cDNA specific for β-actin, and CD36 (Fig. 1-A). Fig. 1-B shows time course of CD36 mRNA expression upon exposure to PrP_106–126_ in BV2 microglia and is expressed as a percentage of CD36 expression in control cells exposed to 100 µM Scr-PrP only.

The mRNA level of CD36 in PrP_106–126_-treated cells was highest at 6 h, when it was 1-fold higher compared to the level in ScrPrP-treated control cells. After 6 h, the mRNA level in PrP106–126-treated cells steadily decreased up to 24 h, but it was still slightly higher than the level in the control (Fig. 1-B).

### 2- PrP_106–126_ induced a CD36-dependent increase of pro-inflammatory cytokines mRNA expression, and IL-1β and IL-6 protein level in microglia

**Figure 2:**
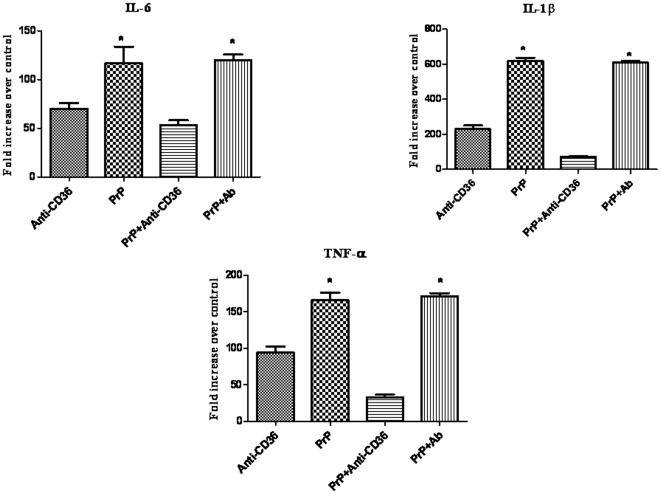
The mRNA expression of proinflammatory cytokines IL-1β, IL-6, and TNF-α, in BV2 microglia treated with PrP_106–126_ in the presence or not of anti-CD36 antibody. Cells were first pre-incubated or not with Anti-CD36 antibody (1 µg/ml) or irrelevant rabbit IgG (Ab) and then treated for 12 hours with 50 µM PrP_106–126_ (PrP). Total mRNA was isolated and reverse-transcribed. The mRNA levels of pro-inflammatory cytokines were measured by quantitative RT-PCR. The mRNA level of each cytokine is expressed as fold increase over control cells which were exposed to PBS only. Data are means ± s.d. of triplicate samples *P, 0.05.

**Figure 3:**
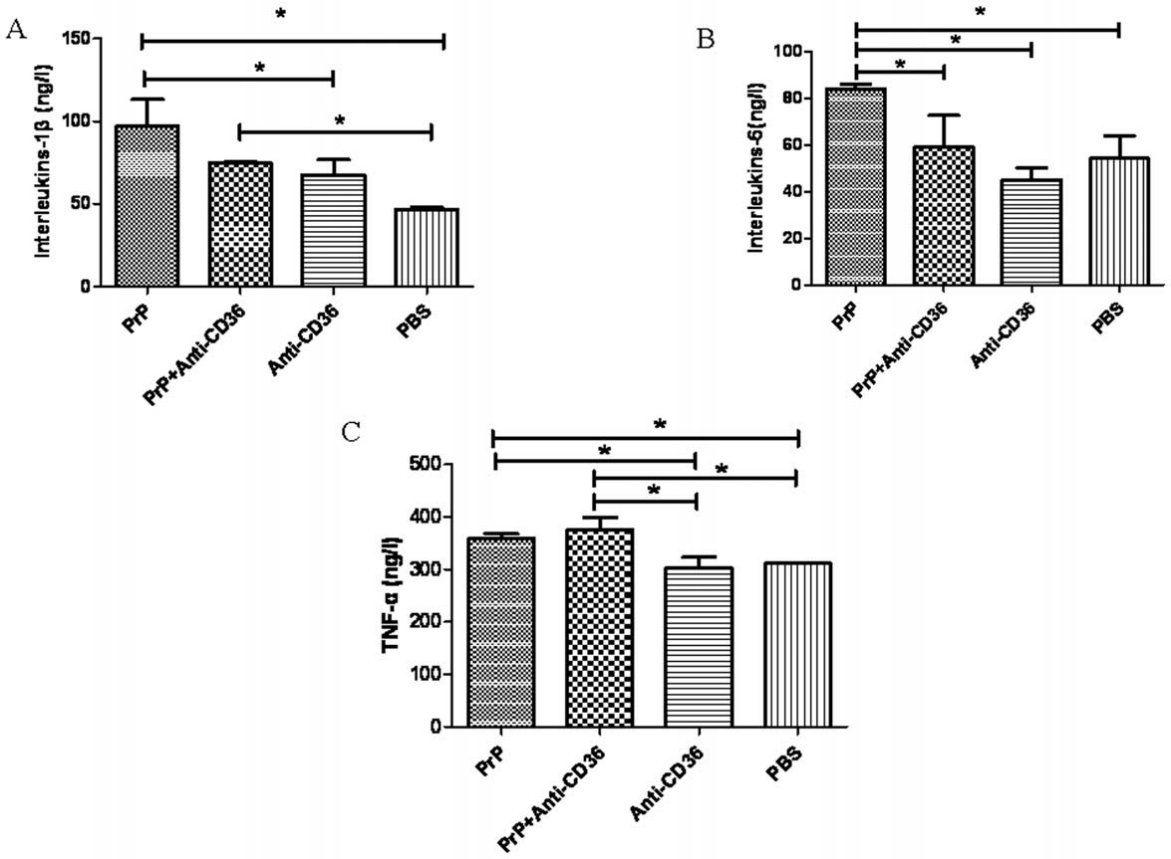
Proinflammatory cytokines (IL-1β, IL-6, and TNF-α) produced by BV2 cells treated with PrP_106–126_ in the presence or not of anti-CD36 antibody. Cells were first pre-incubated or not with Anti-CD36 antibody (1 µg/ml), and then treated for 12 h with PBS or PrP_106–126_ (50 µM). The level of released cytokines was determined in culture supernatants by ELISA as described in Materials and methods. The amount of cytokines (A: IL-1β, B: IL-6 and C: TNF-α) is expressed as ng/l cell supernatant. All data are means ± s.d. of triplicate samples and are representative of an experimental n of 3 or 4, * P<0.05.

The treatment of BV2 cells for 12 h with PrP_106–126_ led to a significant increase in IL-1β, IL-6 and TNF-α at both mRNA and protein levels. For the three cytokines, the increase was statistically significant compared with the level in the cells treated with PBS where the level of cytokines did not significantly change (Fig. 2 and 3).

CD36 blockade with anti-CD36 monoclonal antibody resulted in a significant decrease of the mRNA level of the three cytokines in PrP_106–126_-treated cells. This blocking effect was specific as almost no change was observed on PrP_106–126_-stimulating effect on the mRNA level of the three cytokines in cells pre-incubated with irrelevant rabbit IgG before PrP_106–126_ treatment (Fig. 2).

Pretreatment of BV2 cells with anti-CD36 monoclonal antibody downregulated the PrP_106–126_-induced release of IL-1β and IL-6 as shown by ELISA. The downregulation induced by CD36 blockade was statistically significant for IL6 but not for IL-1β (Fig. 3.A and B). The level of TNF-α released slightly increased after CD36 blockade but was not significantly higher than the level released after PrP_106–126_ treatment (Fig. 3.C). The treatment with anti-CD36 monoclonal antibody alone did not induce any change in the levels of the three cytokines.

Treatment of BV2 cells with Scr-PrP (negative control) or LPS (positive control) led to no change or a significant increase, respectively, in the mRNA and the protein levels of the three cytokines (Data not shown).

### 3- PrP_106–126_ induced a CD36-dependent upregulation of iNOS and NO production

**Figure 4:**
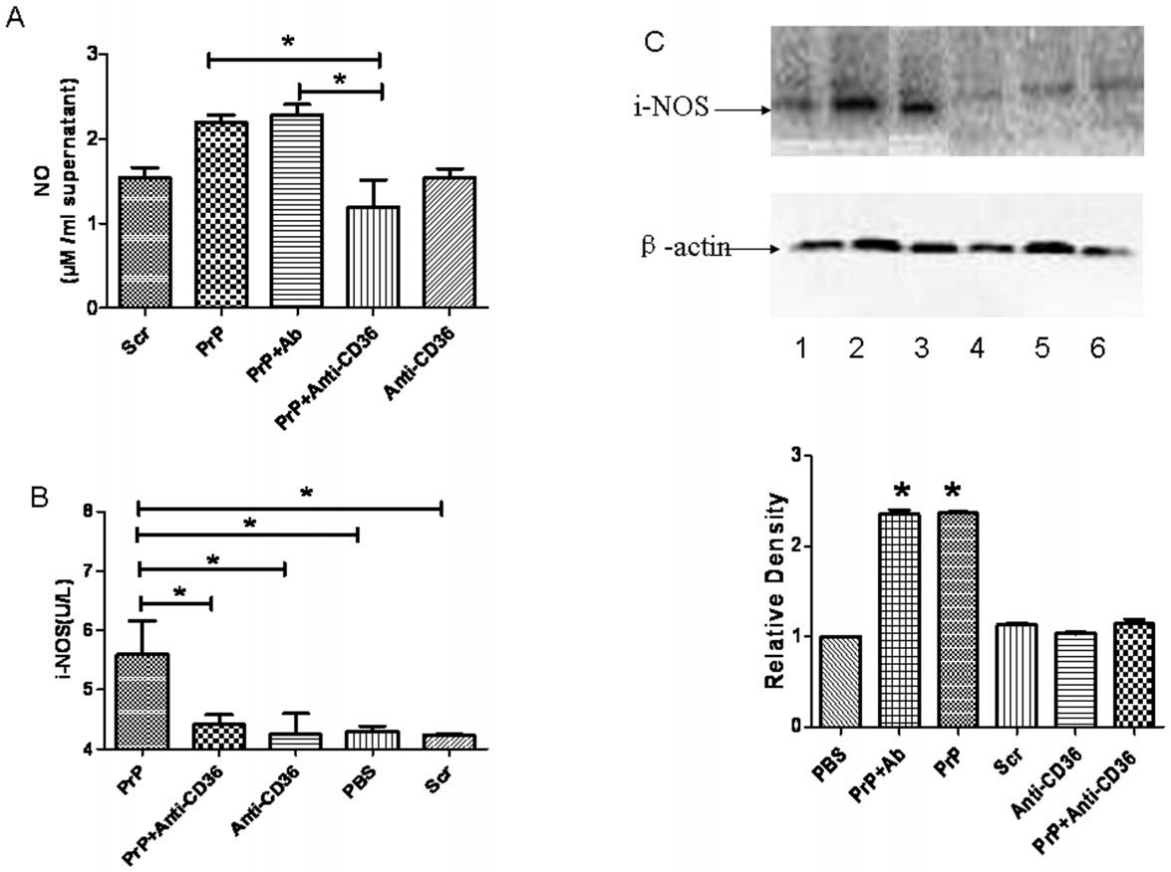
Role of CD36 in PrP_106–126_-induced production of nitric oxide and iNOS expression in BV2 and primary microglia. Cells were first pre-incubated or not with anti-CD36mAb (1 µg/ml) or irrelevant rabbit IgG (Ab) and then treated for 12 hours with PBS, 50 µM PrP_106–126_ (PrP), or scrambled PrP_106–126_ (Scr). A. The level of NO was measured in supernatant from primary microglia culture with nitrite assay as described in Materials and Methods. The amount of nitric oxide is expressed as U/l cell supernatant. B. ELISA was performed on supernatant from culture of BV2 microglia as described in Materials and methods. All data in A and B are means ± s.d. of triplicate samples and are representative of an experimental n of 3 or 4, * P<0.05. C. Cytoplasmic extracts from primary microglia were prepared and immunoblotted with anti-iNOS antibody as described in Materials and Methods. The blot was stripped and reprobed with anti-β-actin antibody to estimate the total amount of protein loaded in gel. Representative blots of iNOS and actin are shown. Bars represent the relative levels of iNOS, compared with β-actin, and were expressed as arbitrary units. Data are the means±S.D of three independent experiments. * P<0.05, significantly different from control cells. 1-PBS, 2–50 µM PrP_106–126_, 3- PrP_106–126_+irrelevant rabbit IgG (Ab), 4- Prpscr (50 µM), 5- Anti-CD36mAb (1 µg/ml), 6- PrP_106–126_+Anti-CD36mAb.

The level of NO in primary microglia treated with 50 µM PrP_106–126_ (2.19±0.09 µM/ml supernatant) for 12 hr was higher than, but not significantly different (P>0.05) from that observed in control cells treated with 50 µM Scr-PrP (1.54±0.12 µM/ml supernatant). CD36 blockade with anti-CD36 monoclonal antibody prior to PrP_106–126_ treatment significantly decreased (P<0.05) NO level (1.19±0.32 µM/ml supernatant). This blocking effect was specific as pretreatment with irrelevant rabbit IgG did not affect PrP_106–126_-induced increase in NO level (Fig. 4-A).

To determine whether the alterations in NO production caused by neurotoxic PrP peptides and CD36 blockade was correlated with alterations in iNOS expression, we examined the level of iNOS in the protein extract from primary microglia and supernatant from BV2 culture exposed to PrP_106–126_ in the presence or not of anti-CD36 antibody.

The results showed that 50 µM PrP_106–126_ treatment resulted in the up-regulation of iNOS in BV2 and primary microglia as shown by ELISA and Western blot analysis (Fig. 4-B and C). Pre-treatment with anti-CD36 antibody followed by PrP_106–126_ stimulation significantly inhibited the PrP_106–126_-induced iNOS up-regulation (Fig. 4-B and C). No iNOS activation was observed in primary microglia treated with anti-CD36 monoclonal antibody or Scr-PrP alone (Fig. 4-C). Again, the blocking effect of anti-CD36 antibody was specific as pretreatment with irrelevant rabbit IgG did not affect PrP106–126-induced increase in iNOS level (Fig. 4-C). Treatment of BV2 cells with LPS (positive control) led to a significant increase in the level of iNOS (Data not shown). The level of i-NOS was significantly higher in cells treated with PrP_106–126_ or irrelevant rabbit IgG plus PrP_106–126_ than in all other examined groups (p<0.05).

### 4- The Blockade of CD36 did not affect PrP_106–126_-induced NF-κB

**Figure 5:**
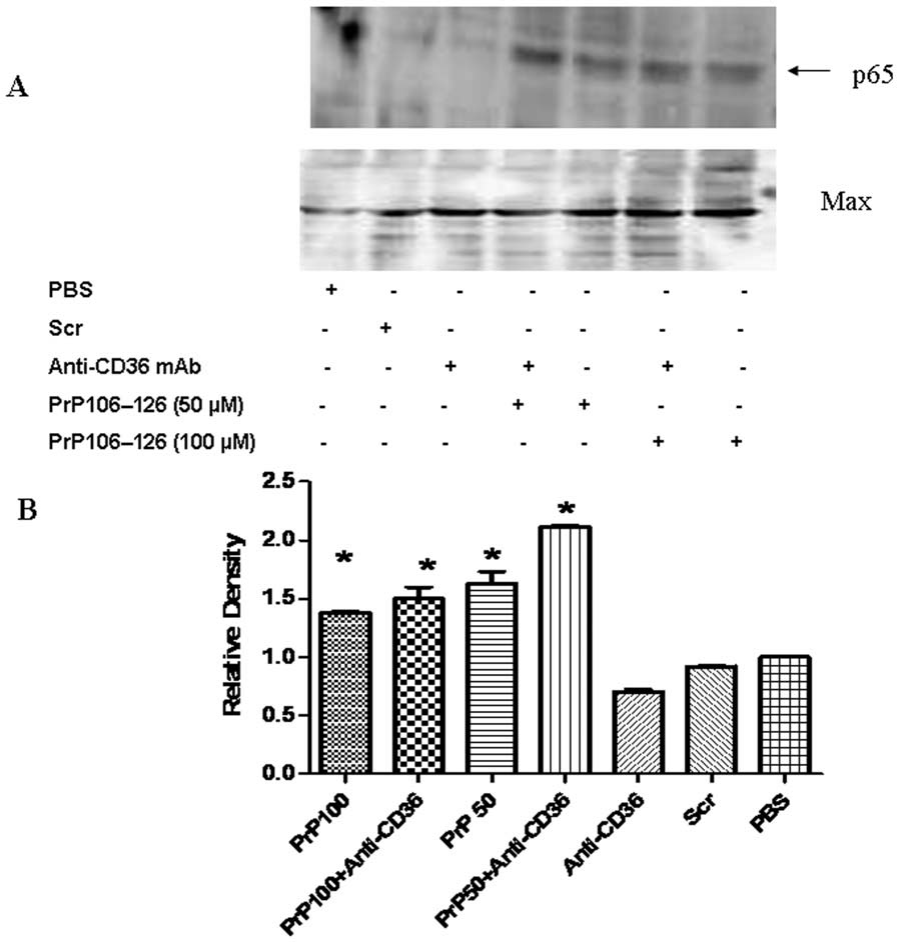
Western blot analysis of p65 nuclear translocation. BV2 microglia were treated or not with two different concentrations of neurotoxic prion peptides (50 µM and 100 µM, respectively) for 24 h. The role of CD36 in NF-KB activation was examined by 1 h-preincubation with anti-CD36 antibody. A. Extracts were prepared and immunoblotted with p65 antibody as described in Materials and Methods. The blot was stripped and reprobed with anti-Max antibody to estimate the total amount of nuclear protein loaded in gel. Representative blots of p65 and Max are shown. B. Bars represent the relative levels of p65, compared with Max, and were expressed as arbitrary units. Data are the means±S.D of three independent experiments. * P<0.05, significantly different from control cells.

The Treatment of BV2 cells with 50 µM or 100 µM PrP_106–126_ led to the activation of NF-κB as indicated by the presence of NF-κB p65 in the nuclear protein extract of the treated cells (Fig. 5). The nuclear translocation of p65 was not affected by CD36 blockade with anti-CD36 antibody (Fig. 5). Even a ten-fold increase of the concentration of anti-CD36 antibody (from 1 µg/ml to 10 µg/ml) to block CD36 did not inhibit PrP_106–126_-induced NF-κB activation (Data not shown). No p65 nuclear translocation was observed in the cells treated with Scr-PrP, PBS, or CD36 monoclonal antibody alone (Fig. 5).

### 5- PrP_106–126_ induced caspase-1 activation even after the Blockade of CD36

**Figure 6:**
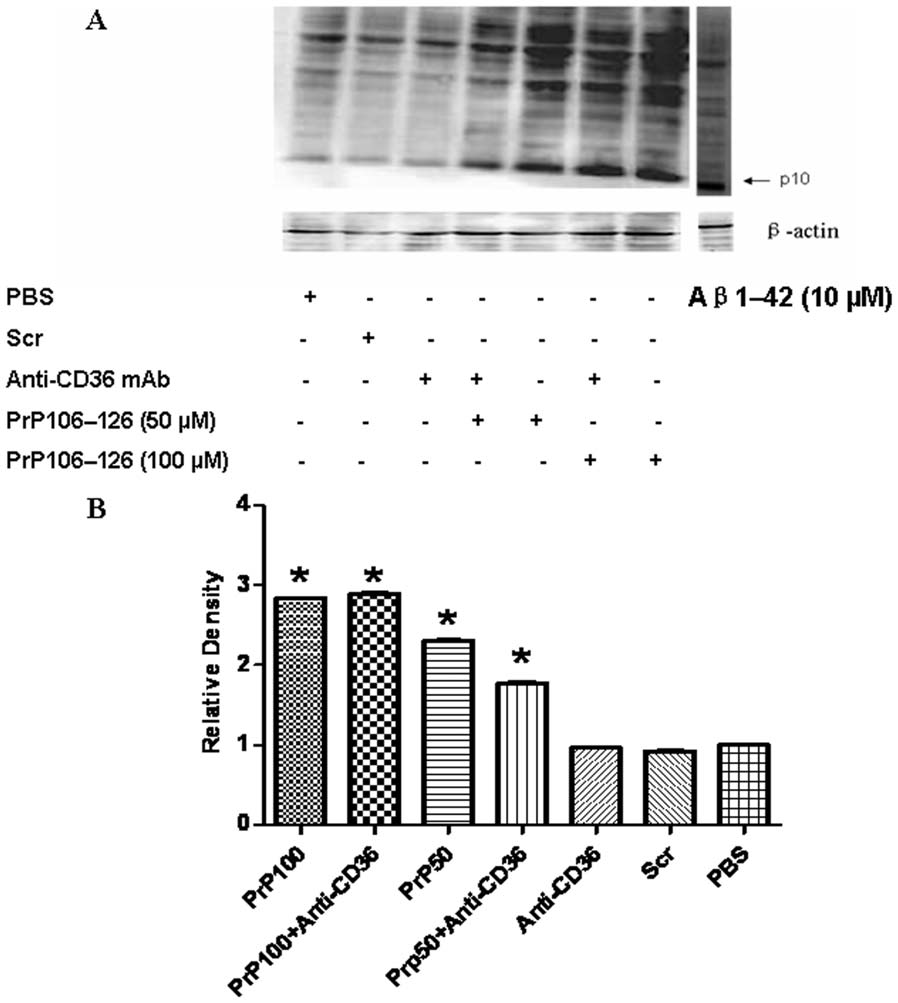
Western blot analysis of caspase-1 cleavage. BV2 microglia were treated or not with two different concentrations of neurotoxic prion peptides (50 µM and 100 µM, respectively) for 24 h. The role of CD36 in caspase1 cleavage was examined by 1 h-preincubation with anti-CD36 antibody. A. Extracts were prepared and immunoblotted with caspase-1 antibody as described in Materials and Methods. The blot was stripped and reprobed with anti-β-actin antibody to estimate the total amount of protein loaded in gel. Representative blots of caspase-1 and β-actin are shown. Extracts of Aβ_1–42_-treated cells were used as positive control. B. Bars represent the relative levels of cleaved caspase-1, compared with β-actin, and were expressed as arbitrary units. Data are the means±S.D of three independent experiments. * P<0.05, significantly different from control cells.

Immunoblot analysis of caspase-1 showed that PrP_106–126_ induced the cleavage of caspase-1 to its active p10 subunit both at 50 µM and 100 µM concentrations (Fig. 6).

Pretreatment with anti-CD36 mAb slightly reduced PrP_106–126_-induced activation of caspase-1 in BV2 cells treated with 50 µM PrP_106–126_, but had no effect on caspase-1 cleavage after tratement with a higher concentration (100 µM) of PrP_106–126_ (Fig. 6). Capase-1 cleavage was not observed in BV2 cells treated with ScrPrP_106–126_, PBS, or CD36 monoclonal antibody alone (Fig. 6). Treatment with 10 µM Amyloid beta, which was used as a positive control, also led to Capase-1 cleavage.

### 6- 6- CD36 bolckade downregulates PrP_106–126_ induced Fyn activity

**Figure 7:**
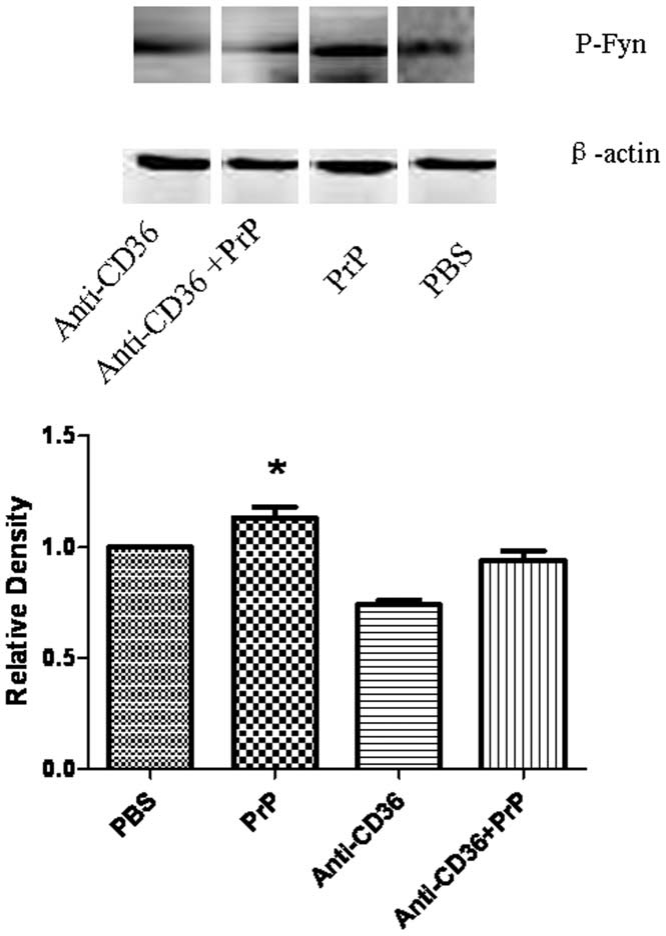
Western blot analysis of p-Fyn. BV2 microglia were treated or not with neurotoxic prion peptides (100 µM) for 24 h after 1 h-preincubation with anti-CD36 mAb. The role of Fyn in CD36 signaling was examined by assessing Fyn phosphorylation. A. Extracts were prepared and immunoblotted with phospho-Fyn (p-Fyn) antibody as described in Materials and Methods. The blot was stripped and reprobed with anti-β-actin antibody to estimate the total amount of protein loaded in gel. Representative blots of p-Fyn and β-actin are shown. B. Bars represent the relative levels of cleaved p-Fyn, compared with β-actin, and were expressed as arbitrary units. Data are the means±S.D of three independent experiments. * P<0.05, significantly different from control cells.

Immunoblot analysis of phospho-Fyn (p-Fyn) showed that treatment with 100 µM PrP_106–126_ led to Fyn activation as indicated by the increase in the level of p-Fyn. The level of p-Fyn was higher in cells treated with PrP_106–126_ than in those treated with PBS. Blockade of CD36 with anti-CD36 monoclonal antibody significantly reduced PrP_106–126_-induced increase in the level of p-Fyn (Fig. 7).

## Discussion

Microglia activation involves multiple pathways that result in morphological changes, proliferation and release of bioactive substances that play an important role in the onset and progression of neurodegenerative diseases such as Alzheimer's disease and prion diseases [5]. Several studies have reported that PrP synthetic peptides induce microglial activation [21], [22], [23], [24]. The identification of cell surface molecules that mediate the PrP synthetic peptides interaction with microglia is of great importance as it represents the first point of intervention in the events leading to the pathophysiology of prion diseases. Our approach to investigate the role of CD36 in the PrP_106–126_-induced microglia activation derived from consideration of its well-documented role in other protein misfolding-diseases such as Alzheimer's disease.

We started by examining the effect of exposure to PrP_106–126_ (100 µM) on the expression of *CD36* gene in BV2 cells. The mRNA level of *CD36* increased significantly after 6 h of exposure to the prion peptides and steadily decreased during the time interval between 12 and 24 h. The general pattern of the expression of CD36 mRNA observed in this experiment is similar to that that we have reported in a recent study [25]. In that study, the concentration of PrP_106–126_ was lower (50 µM) and the increase in CD36 started earlier (at 3 h) but started to decrease at 6 h. Our result is consistent with many reports in the literature supporting the idea of inducible overexpression of CD36 in microglia after exposure to amyloid fibril [26], [27], and is compatible with a possible participation of CD36 in PrP_106–126_-induced microglial activation.

Our approach then consisted of examining the effect of blocking CD36 by monoclonal antibody on several parameters of microglial activation. The adoption of this approach was motivated by the fact that CD36 tends to act as a component of a cell surface receptor complex that mediates the binding of microglia to fibrillar proteins or other ligands [11], [28], [29]. We hypothesized that by blocking CD36 with its monoclonal antibody we could competitively inhibit the formation of the receptor complex and this would reveal its role in the process of microglial activation upon stimulation with prion peptides.

Proinflammatory cytokines are important effector molecules that act as proinflammatory factors and are thought to paly an important role in neurodegeneration. There have been conflicting reports of the effect of prion peptides on the level of inflammatory cytokines in microglia [23], [30], [31]. In this study, we showed that PrP_106–126_ induced a significant increase in the expression of IL-1β, IL-6 and TNF-α both at mRNA and protein levels. Interestingly, pretreatment with anti-CD36 monoclonal antibody significantly abrogated the PrP_106–126_-induced increase in the mRNA expression of the three cytokines. At the protein level, however, the blocking effect of anti-CD36 antibody was maintained only for IL-6, IL-1β, albeit not significantly for the latter. In contrast, CD36 blockade did not alter PrP_106–126_-induced increase in the protein level of TNF-α although it significantly downregulated the increase in its mRNA transcription induced by PrP_106–126_ treatment. These results suggest that CD36 blockade did not downregulate TNF-α mRNA transcription to the extent that would affect its protein synthesis and support a central role of CD36 in the signaling that leads to the up-regulation of IL-6, IL-1β in microglia upon exposure to PrP_106–126_.

It is well established that stressful conditions can trigger the expression of iNOS, which can generate NO from Larginine [32]. In this study, we reported that PrP_106–126_ induced an increase in iNOS level and NO secretion in primary microglia. This is in line with other reports that almost invariably reported the upregulation of iNOS and release of NO in macrophages and microglia exposed to neurotoxic prion peptides [9], [30], [31]. Moreover, we showed that CD36 blockade significantly abrogated the effect of PrP_106–126_ treatment on iNOS expression and NO production. These results are consistent with previous reports showing that CD36 mediates free radical production in many neuroinflammatory conditions including Alzheimer disease and cerebral ischemia [11], [33], [34], and support a key role of CD36 in prion diseases-associated oxidative stress by triggering iNOS up-regulation and NO production.

Several lines of evidence indicate that NF-κB activation is crtitical for the induction of iNOS and the upregulation of inflammatory cytokines such as IL-1β and IL-6 [35], [36], [37], [38]. Moreover, the activation of NF-κB in macrophages and microglia exposed to neurotoxic prion peptides is well documented [30], [39], [40]. NF-κB activation was also linked to CD36 signaling [41], [42]. We therefore examined the effect of CD36 blocking on PrP_106–126_-induced NF-κB activation. The observed results showed nuclear translocation of p65 in PrP_106–126_-treated cells even in the case of anti-CD36 monoclonal antibody pretreatment. This finding may account for why the release of TNF-α in the treated cells was not affected by CD36 blockade. However, keeping in mind that a wide variety of signals emanating from antigen receptors, pattern-recognition receptors, receptors for the members of TNF and IL-1 cytokine families, and others induce differential activation of NF-κB, this result is not conclusive and does not rule out the possibilty that CD36 blockade may inhibit microglial activation by interfering with NF-κB activation. We can speculate, for example, that PrP_106–126_ leads to the activation of NF-κB through several pathways and that, if any, only one or some of these pathways are CD36-mediated.

We also examined the effect PrP_106–126_ treatment on caspase-1 activation. The result showed that PrP_106–126_ stimulated capase-1 cleavage and that CD36 blockade did not interfere with this activation although a slight reduction of caspase-1 cleavage was observed after a moderate PrP_106–126_ treatment. This suggests that CD36 may not play a central role in PrP_106–126_-induced capase-1 activation. Caspase-1 is an inflammatory caspase, whose main substrates are cytokines that are crucial to the inflammatory response such IL-1β and IL-18. Caspase-1 is present as an inactive proform in the cytoplasm and it is activated by proteolytic self-processing. Several multimolecular proteins complexes, referred to as inflammasomes, have been identified as caspase-1 activators [43], [44], [45]. To our knowledge, this is the first time an association between neurotoxic prion peptides and activation of caspase-1 in microglia is reported. This finding suggests that inflammsome activation may be involved in neurotoxic prion peptides-induced inflammation and, if confirmed by further studies, would add prion diseases to a long list of diseases and inflammatory conditions that have been associated with inflammasome.

Caspase-1 activation leads to post-translational processing and release of the mature, biologically active forms of IL-1β and IL-18, which are potent mediators of inflammation, being responsible for a variety of effects associated with host responses to microbial invasion and tissue damage [43]. As mentioned above, PrP_106–126_ induced IL-1β upregulation and capase-1 activation. However, CD36 blockade resulted only in the inhibition of PrP_106–126_-induced upregulation of IL-1β without a significant effect on capase-1 activation, which suggests that CD36 mediates PrP_106–126_-induced upregulation of IL-1β through a capase-1-independent pathway. Accordingly, the fact that CD36 blockade significantly downregulated mRNA expression of IL-1β in PrP_106–126_-treated cells supports a direct involvement of CD36-mediated signaling at the level of mRNA transcription of IL-1β in microglia exposed to neurotoxic prion peptides.

Finally, we examined the effect of PrP_106–126_ treatment on Fyn phosphorylation. Fyn is a membrane associated non-receptor protein tyrosine kinase that belongs to the Src family of cytoplasmic tyrosine kinase [46]. Fyn is expressed predominately in tissues of neuronal and hematopoietic origin and has been shown to be involved in CD36-mediated signaling [47]. The decrease in p-Fyn level observed after CD36 blockade in PrP_106–126_ treated microglia suggests that the participation of CD36 in the interaction between PrP_106–126_ and microglia may be mediated by Src tyrosine kinases. This is supported by preliminary results from a research work in our lab on CD36 signaling during PrP_106–126_-induced micrglial activation, which is currently underway, and which showed that Src tyrosine kinase inhibitor, PP2, can significantly downregulate PrP_106–126_-induced i-NOS upregulation in BV2 micrglia.

In conclusion, we have shown that CD36 is involved in PrP_106–126_-induced microglial activation and that neurotoxic prion peptides can induce caspase-1 activation in microglia. These findings unveil a previously unrecognized role of CD36 as a surface molecule involved in neurotoxic prion peptides-microglia interactions and raise the possibility of inflammasome involvement in the pathogenesis of prion diseases, thus providing new insights into the mechanisms underlying the activation of microglia by neurotoxic prion peptides. Although more studies are needed to confirm and explore these initial findings, our study identified a potential molecular target for the treatment of prion diseases and provides perspectives for new therapeutic strategies for prion diseases by modulation of CD36 signaling.
