# Correction: CD36 Participates in PrP_106–126_-Induced Activation of Microglia

**DOI:** 10.1371/annotation/f900d37b-3d36-4551-8680-cecf4bd1418d

**Published:** 2012-02-29

**Authors:** Mohammed Kouadir, Lifeng Yang, Rongrong Tan, Fushan Shi, Yun Lu, Siming Zhang, Xiaomin Yin, Xiangmei Zhou, Deming Zhao

There was an error in the published funding statement. The correct funding statement is, "This work was supported by the Ministry of Agriculture, Key Program, China (Project No. 2009ZX08008-010B and No. 2009ZX08007-008B), The Natural Science Foundation of China (Project No. 31001048, No. 30972164, and No. 30871854), Beijing Natural Science Foundation (Project No. 6101002), the Program for Cheung Kong Scholars and Innovative Research Team in University of China (No. IRT0866). The funders had no role in study design, data collection and analysis, decision to publish, or preparation of the manuscript."

